# Anomalous strain energy transformation pathways in mechanical metamaterials

**DOI:** 10.1098/rspa.2019.0041

**Published:** 2019-06-05

**Authors:** Eduard G. Karpov, Larry A. Danso, John T. Klein

**Affiliations:** Department of Civil and Materials Engineering, University of Illinois, Chicago, IL 60607, USA

**Keywords:** transformation mechanics, mechanical metamaterials, entropy

## Abstract

This discussion starts with a mechanics version of Parseval's energy theorem applicable to any discrete lattice material with periodic internal structure: a microtruss, grid, frame, origami or tessellation. It provides a simple relationship between the strain energy volumetric/usual and *spectral* distributions in the reciprocal space. The spectral energy distribution leads directly to a spectral entropy of lattice deformation (Shannon's type), whose variance with a material coordinate represents the decrease of information about surface loads in the material interior. Spectral entropy is also a basic measure of complexity of mechanical responses of metamaterials to surface and body loads. Considering transformation of the energy volumetric and spectral distributions with a material coordinate pointed away from a surface load, several interesting anomalies are seen even for simple lattice materials, when compared to continuum materials. These anomalies include selective filtering of surface Raleigh waves (sinusoidal pressure patterns), Saint–Venant effect inversion illustrated by energy spectral distribution contours, occurrence of ‘hiding pockets’ of low deformation, and redirection of strain energy maximum away from axis of a concentrated surface load. The latter phenomenon can be significant for impact protection applications of mechanical metamaterials.

## 1. Introduction

The science of mechanical metamaterials emerged at the interface of applied mechanics and materials engineering, inspired by the earlier research in optics, acoustics and electromagnetism [1–11], to explore opportunities for materials with exotic mechanical properties rarely found in the nature. The modern research in mechanical metamaterials encompasses design, theoretical, experimental and computational studies of material systems with negative Poisson's ratio [12–14], negative bulk modulus [15,16], negative longitudinal stiffness and compliance [17–21], reverse Saint–Venant's effect [22] and other anomalous elastic properties. Interesting properties of the metamaterials are defined mostly by their internal structure, topology and geometric architecture rather than chemical composition. Design of metamaterials properties by means of stiffness modulations and application of large amounts of deformation has been discussed as well [23,24]. Internal structure design of lattice materials, foams, granular materials, origamis kirigamis, tessellations, tensegrities and minimal surface may also lead to a range of *functional* properties, such as reconfigurability, multistability, polymorphism, symmetry breaking, deformation and strain energy reprogramming [19–22,25–32].

Strain energy control, reprogramming and redistribution [22,31,32] is one topic, where few studies have been done, although mechanical energy bending and deflection away from an obstacle is a typical challenge in the closely related field of acoustical metamaterials [8,9]. One possible reason is a lack of analytical tools, or a standardized formulation that could unveil a full potential of mechanical materials for this type of functionalities. Polymorphic mechanical metamaterials may well perform as superdampers [16,21]. Moreover, their unusual quasi-static performances could complement acoustical metamaterials for fast *aperiodic* impact loads, whose frequency spectra extend far beyond any reasonable acoustical metamaterial's bandgap. Such an impact load then could be more efficiently damped in a supersonic regime, by controlling instantaneous strain energy distributions in the material even before any oscillatory process is established. Therefore, it is important to understand, if mechanical metamaterials can provide a mechanism to control the strain energy distribution and transformation. Harnessing these mechanisms would also suggest opportunities to employ *spatial profiles* of impact loads [22,31], in addition to their frequency spectra, for highly efficient damping performance.

The first objective of this work is to discuss some novel analytical tools and concepts enabling a systematic analysis of strain energy transformation in any periodic material system: lattice associate cell and a universal strain energy representation, Raleigh decay spectrum, strain energy spectral distribution and a *spectral theorem* that connects the volumetric and spectral distributions, and *Shannon's entropy* of lattice deformation. Energy spectral theorem is analogous to Parseval's theorem, written for squared data points, $x_{m}$, and their Fourier transform, $\tilde{x}(q)$,

1.1 $$\frac{1}{M}\sum_{m=0}^{M-1}|x_{m}{|}^{2}=\sum_{q}|\tilde{x}(q){|}^{2}$$

and

1.2 $$\tilde{x}(q)=\sum_{m=0}^{M-1}x_{m}{\text{e}}^{-\text{i}qm},\;q=\frac{2\pi \mu }{M},\;\mu =0,1,\ldots ,M-1.$$

The squared Fourier transform represents power spectral density of a signal

1.3 $$\tilde{W}(q)=|\tilde{x}(q){|}^{2}.$$

Shannon's spectral entropy of the signal reads

1.4 $$\begin{array}{rl} H & =\sum_{q}\tilde{w}(q)\;\ln\;\tilde{w}(q)\\ \;\text{and}\;\tilde{w}(q) & =\frac{\tilde{W}(q)}{\sum_{q}\tilde{W}(q)}, \end{array}\}$$

and it is interpreted as purity of the signal or used to distinguish between artificial (low-entropy) and natural (high-entropy) signals. A *mechanical*, strain energy version of Parseval's theorem (1.1) was shown earlier in [32] for the case of elastic continuum (homogeneous materials), and, here, we demonstrate a discrete version of this theorem and information entropy calculation for mechanics of architectured materials.

**Figure 1.**
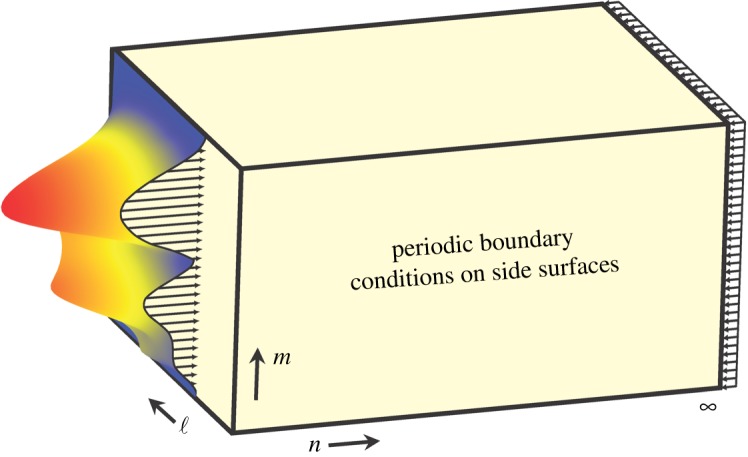
Generic problem statement with a material sample subjected to an interesting quasi-static load/pressure pattern at the left and, a smooth reaction at the right end, and periodic boundary conditions on other faces. (Online version in colour.)

The second objective of this work is a demonstration of utility of these new analytical tools for a systematic analysis of strain energy transformation behaviour of materials with periodic internal structure. Qualitative shapes of the Raleigh decay spectrum determine how the material translates the instantaneous strain energy of deformation between two points in space. At varying design parameters, the material may demonstrate only a few qualitatively different shapes/classes of its Raleigh spectrum, corresponding to several distinct and well-defined energy transformation behaviours. A systematic understanding of these behaviours can be gained considering a generic problem statement of figure 1 type, where the sample is subjected to an interesting load pattern on the let end and to a smooth reaction at right end. A triangular lattice discussed here will have two distinct Raleigh spectra, and an X-braced lattice three. One of them can provide selective surface pressure *blockage*, and another leads to strain energy *redirection* out of the axis of a concentrated surface load. Such functionalities of mechanical metamaterials can be interesting for a range of military, civil and mechanical engineering applications.

## 2. Strain energy distribution in periodic materials

Analysis of the strain energy distribution in engineered materials with periodic internal structure (lattices, tensegrities, tessellations and origamis) can be rather involved, compared to the continuum solids, due to the fundamental non-local nature of discrete material systems. Indeed, in discrete materials, internal elastic forces in a point of interest always depend on material deformation in some *finite* region surrounding that point. This non-locality imposes difficulty defining a representative volume of the lattice where the strain energy can be universally written. In *simple* lattices and most tessellations, a closed unit cell may be defined, representing a rigid polyhedron or a group of polyhedrons that repeats in space to mosaic the entire material sample. This repetition may occur in one, two or three directions, not necessarily mutually orthogonal, for the chain-like, plate-like (two-dimensional) and volumetric (three-dimensional) lattices. The strain energy is then a sum of the unit cell energies

2.1 $$W_{\text{total}}=\sum W_{\text{u}.\text{c}.}$$

**Figure 2.**
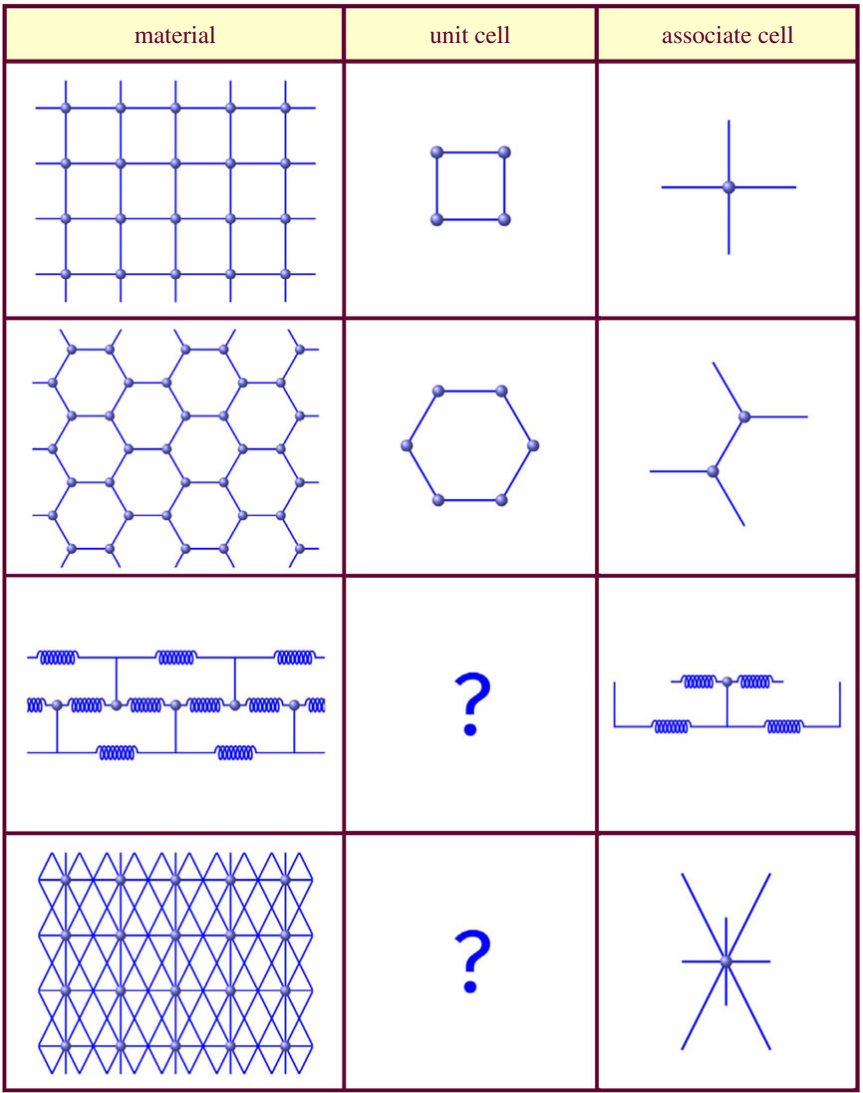
Periodic lattice materials. A repetitive unit cell cannot be defined in openwork materials with higher non-locality without element cutting. On the contrary, an associate can be introduced for any periodic material. (Online version in colour.)

This approach fails when a closed unit cell cannot be defined and the structural links may protrude out of a repetitive cell, as in tensegrities and ‘braided’ lattices (figure 2). We call such materials *highly non-local* [22] or essentially non-local. In this case, a unit cell definition requires imaginary cutting or certain structural elements, and it becomes more convenient to introduce an *associate cell* instead. An associate cell is the smallest part of the periodic material, containing a minimal group of distinct structural nodes, and all structural elements connected to these nodes. Note that there is only one type of periodic nodes in all figure 2 examples, except for the hexagonal lattice shown in the second row. Here, two distinct types of nodes exist; the first type has horizontal members attached on the right side, and the second type has them on the left. As a result, the associate cell shown in second row of figure 2 contains two such nodes and all the members directly connected to these nodes.

A great feature of this approach is that an associate cell exists for *any* periodic material, without element cutting. Moreover, for materials with one distinct type of periodic nodes and only pair-wise elastic elements, the strain energy is simply a half sum of the associate cell energies

2.2 $$W_{\text{total}}=\frac{1}{2}\sum W_{\text{a}.\text{c}.}=\sum_{n,m,l}W_{nml}.$$

Here, a triplet of integer indices, (n,m,l), is assigned to every periodic node in the material volume, $W_{\text{a}.\text{c}.}$ is strain energy of an associate cell centred at the node (n,m,l). The three-dimensional array $W_{nml}$ in (2.2) is understood as a discrete function of the integer indices, and it represents a *strain energy volumetric distribution* from node to node in the lattice.

A wide range of openwork rigid lattice materials and tensegrities can be accurately described with a model, where every structural element is simply a pair-wise elastic link with no rotational degrees of freedom, such as a metal bar, spring, rubber band, steel cable, etc. Accuracy of the bar-based model of periodic materials can be insufficient for rigid-joined lattices, periodic tessellations and origamis. Here, models using Euler–Bernoulli and Timoshenko beams, or even three-dimensional finite elements can be used alternatively. In all cases, a structural stiffness matrix can be evaluated for one associate cell, and it will contain the basic matrices of force constants, ${\text{K}}_{n-n^{\prime }m-m^{\prime }l-l^{\prime }}$, in the rows corresponding to the degrees of freedom of the node (n,m,l). More details on these matrices can be found elsewhere [22,31,33–35], and some examples are also shown here in §6. They describe intensity of *direct* elastic interactions of a periodic node (n,m,l) with its neighbours $\left(n^{\prime },m^{\prime },l^{\prime }\right)$, so that any external forces or couple moments acting at (n,m,l) can be written as

2.3 $${\text{f}}_{nml}=\sum_{n^{\prime },m^{\prime },l^{\prime }}{\text{K}}_{n-n^{\prime }m-m^{\prime }l-l^{\prime }}{\text{d}}_{n^{\prime }m^{\prime }l^{\prime }}.$$

Here, f is a vector of the force and/or moment components, and d is a vector of kinematical degrees of freedom (displacements and rotations) of the structural nodes. This equation represents a governing equation of equilibrium of an arbitrary periodic material [22,31,33–35]. Importantly, every term in the right-hand side of (2.3) is an internal force at $\left(n^{\prime },m^{\prime },l^{\prime }\right)$ up to a sign inversion. A total mechanical work done by these forces gives energy of deformation stored in the associate cell at the node (n,m,l), see equation (2.2), and therefore we may write the *strain energy volumetric distribution*

2.4 $$W_{nml}=\frac{1}{2}W_{\text{a}.\text{c}.}=\frac{1}{2}\sum_{n^{\prime },m^{\prime },l^{\prime }}{\text{d}}_{n^{\prime }m^{\prime }l^{\prime }}^{*}{\text{K}}_{n-n^{\prime }m-m^{\prime }l-l^{\prime }}{\text{d}}_{n^{\prime }m^{\prime }l^{\prime }},$$

where ${\text{d}}^{*}$ is a conjugate transpose of the displacement vector.

## 3. Strain energy spectral theorem

The energy representation (2.4) allows writing a distribution of the strain energy in the direction of a chosen spatial index by summing up (2.4) over two other indices

3.1 $$\Pi_{n}=\sum_{m,l}W_{nml}=\frac{1}{2}\sum_{m,l}\sum_{n^{\prime },m^{\prime },l^{\prime }}{\text{d}}_{n^{\prime }m^{\prime }l^{\prime }}^{*}{\text{K}}_{n-n^{\prime }m-m^{\prime }l-l^{\prime }}{\text{d}}_{n^{\prime }m^{\prime }l^{\prime }}.$$

This discrete function shows how the strain energy is distributed among parallel layers of the material, where each layer contains all the *n*-numbered nodes. With this form, derivation of a mechanical version of Parseval's spectral theorem (1.1) for lattice materials becomes straightforward. Indeed, we may replace the displacement vectors in (3.1) with the inverse Fourier transforms

3.2 $${\text{d}}_{nml}=\frac{1}{ML}\sum_{q,r}{\tilde{\text{d}}}_{n}(q,r){\text{e}}^{\text{i}qm}{\text{e}}^{\text{i}rl}$$

and

3.3 $${\text{d}}_{nml}^{*}=\frac{1}{ML}\sum_{q^{\prime },r^{\prime }}{\tilde{\text{d}}}_{n}^{*}(q^{\prime },r^{\prime }){\text{e}}^{-\text{i}q^{\prime }m}{\text{e}}^{-\text{i}r^{\prime }l},$$

of their own partial Fourier images

3.4 $${\tilde{\text{d}}}_{n}(q,r)=\sum_{m,l}{\text{d}}_{nml}{\text{e}}^{-\text{i}qm}{\text{e}}^{-\text{i}rl}$$

and

3.5 $${\tilde{\text{d}}}_{n}^{*}(q^{\prime },r^{\prime })=\sum_{m,l}{\text{d}}_{nml}^{*}{\text{e}}^{\text{i}q^{\prime }m}{\text{e}}^{\text{i}r^{\prime }l}.$$

Here *q* and *r* are fractional wavenumber indices (q=2πμ/M, r=2πℓ/L), and μ and ℓ are integer wavenumber indices (μ=0,1,…,M−1, ℓ=0,1,…,L−1). *M* and *L* are the total numbers of the Fourier harmonics, or ranges of the spatial indices *m* and *l* in a domain with periodic boundary conditions, where ${\text{d}}_{nml}={\text{d}}_{nm+Ml+L}$ for any *m* and *n*. Orthogonality of the Fourier modes

3.6 $$\begin{array}{rl} \sum_{m}{\text{e}}^{-\text{i}qm}{\text{e}}^{\text{i}q^{\prime }m} & =M\delta_{qq^{\prime }}\\ \;\text{and}\;\sum_{l}{\text{e}}^{-\text{i}rl}{\text{e}}^{\text{i}r^{\prime }l} & =N\delta_{rr^{\prime }}, \end{array}\}$$

where δ is the Kronecker delta, removes the sums over $q^{\prime }$ and $r^{\prime }$ in (3.1) and (3.3) and gives an alternative form of the strain energy distribution

3.7 $$\Pi_{n}=\frac{1}{2ML}\sum_{q,r}\sum_{n^{\prime }}{\tilde{\text{d}}}_{n^{\prime }}^{*}(q,r){\tilde{\text{K}}}_{n-n^{\prime }}(0,0){\tilde{\text{d}}}_{n^{\prime }}(q,r).$$

Here, the matrices

3.8 $${\tilde{\text{K}}}_{n}(0,0)=\sum_{ml}{\text{K}}_{nml}$$

are interpreted as a Fourier transform (1.2) of the matrices ${\text{K}}_{nml}$,

3.9 $${\tilde{\text{K}}}_{n}(q,r)=\sum_{m,l}{\text{K}}_{nml}{\text{e}}^{-\text{i}qm}{\text{e}}^{-\text{i}rl},$$

evaluated at q=r=0. Finally, we can compare (3.1) and (3.7) and write the *spectral theorem* of mechanics of a discrete periodic elastic medium

3.10 $$\begin{array}{rl} \Pi_{n} & =\sum_{m,l}W_{nml}=\sum_{q,r}{\tilde{W}}_{n}(q,r), \end{array}$$

3.11 $$\begin{array}{rl} W_{nml} & =\frac{1}{2}\sum_{n^{\prime },m^{\prime },l^{\prime }}{\text{d}}_{n^{\prime }m^{\prime }l^{\prime }}^{*}{\text{K}}_{n-n^{\prime }m-m^{\prime }l-l^{\prime }}{\text{d}}_{n^{\prime }m^{\prime }l^{\prime }} \end{array}$$

3.12 $$\begin{array}{rl} \;\text{and}\;{\tilde{W}}_{n}(q,r) & =\frac{1}{2ML}\sum_{n^{\prime }}{\tilde{\text{d}}}_{n^{\prime }}^{*}(q,r){\tilde{\text{K}}}_{n-n^{\prime }}(0,0){\tilde{\text{d}}}_{n^{\prime }}(q,r). \end{array}$$

While the function $W_{nml}$ of (3.1) represents a volumetric distribution of the strain energy, the function ${\tilde{W}}_{n}(q,r)$ of (3.11) is equally interesting. It is a *spectral distribution* of the strain energy (among the Fourier modes) at different spatial locations, *n*, in the material interior.

There are two useful remarks regarding the spectral theorem ((3.10) and (3.12)). First, we may rewrite it using the normalized energy distributions

3.13 $$\begin{array}{rl} \sum_{m,l}w_{nml} & =\sum_{q,r}{\tilde{w}}_{n}(q,r)=1\;(\text{at any}\;n), \end{array}$$

3.14 $$\begin{array}{rl} w_{nml} & =\frac{W_{nml}}{\Pi_{n}} \end{array}$$

3.15 $$\begin{array}{rl} \;\text{and}\;{\tilde{w}}_{n}(q,r) & =\frac{{\tilde{W}}_{n}(q,r)}{\Pi_{n}}. \end{array}$$

Second, a full Fourier transform of the displacement vectors

3.16 $$\hat{\text{d}}(p,q,r)=\sum_{n,m,l}{\text{d}}_{nml}{\text{e}}^{-\text{i}(pn+qm+rl)},$$

could also be used to give the following equality:

3.17 $$W_{\text{total}}=\sum_{n,m,l}W_{nml}=\sum_{p,q,r}\hat{W}(p,q,r)$$

and

3.18 $$\hat{W}(p,q,r)=\frac{1}{2NML}{\hat{\text{d}}}^{*}(p,q,r)\hat{\text{K}}(0,0,0)\hat{\text{d}}(p,q,r),$$

where $\hat{W}(p,q,r)$ is an overall spectral distribution of the strain energy in a material sample. However, for many practical problems related to mechanical metamaterials, it is interesting to see how the strain energy spectral distribution translates into the material volume, or how the shape of the dependence of $\tilde{W}$ on *q* and *r* transforms at a varying spatial index *n*. Therefore, the result (3.10) and (3.12) or (3.13) and (3.15) is more interesting in practice.

For *plate-like lattice models* governed by a two-dimensional version of the governing equation (2.3)

3.19 $${\text{f}}_{nm}=\sum_{n^{\prime },m^{\prime }}{\text{K}}_{n-n^{\prime }m-m^{\prime }}{\text{d}}_{n^{\prime }m^{\prime }},$$

the spectral theorem reads

3.20 $$\begin{array}{rl} \Pi_{n} & =\sum_{m}W_{nm}=\sum_{q}{\tilde{W}}_{n}(q), \end{array}$$

3.21 $$\begin{array}{rl} W_{nm} & =\frac{1}{2}\sum_{n^{\prime }m^{\prime }}{\text{d}}_{n^{\prime }m^{\prime }}^{*}{\text{K}}_{n-n^{\prime }m-m^{\prime }}{\text{d}}_{n^{\prime }m^{\prime }}, \end{array}$$

3.22 $$\begin{array}{rl} {\tilde{W}}_{n}(q) & =\frac{1}{2M}\sum_{n^{\prime }}{\tilde{\text{d}}}_{n^{\prime }}^{*}(q){\tilde{\text{K}}}_{n-n^{\prime }}(0){\tilde{\text{d}}}_{n^{\prime }}(q), \end{array}$$

or, in terms of the normalized energy densities

3.23 $$\begin{array}{rl} \sum_{m}w_{nm} & =\sum_{q}{\tilde{w}}_{n}(q)=1\;(\text{at any}\;n), \end{array}$$

3.24 $$\begin{array}{rl} w_{nm} & =\frac{W_{nm}}{\sum_{m}W_{nm}} \end{array}$$

3.25 $$\begin{array}{rl} \;\text{and}\;{\tilde{w}}_{n}(q) & =\frac{{\tilde{W}}_{n}(q)}{\sum_{q}{\tilde{W}}_{n}(q)}. \end{array}$$

The partial Fourier transforms in (3.19) are simply

3.26 $$\begin{array}{rl} {\tilde{\text{d}}}_{n}(q) & =\sum_{m}{\text{d}}_{nm}{\text{e}}^{-\text{i}qm} \end{array}$$

3.27 $$\begin{array}{rl} {\tilde{\text{K}}}_{n}(q) & =\sum_{m}{\text{K}}_{nm}{\text{e}}^{-\text{i}qm} \end{array}$$

We note that the cost of computation of the strain energy spectral distribution ((3.12) and (3.22)) is low, being a linear function of *NML* or *NM*, the total number of unit cells in the lattice, as typical for all Fourier transform based methods [34,35]. Such methods essentially block-diagonalize the original governing equations or decouple any repetitive degrees of freedom in the lattice structure.

## 4. Spectral entropy of deformation

The strain energy spectral distribution ((3.12) and (3.22)) describes how periodic materials translate energy of deformation into the reciprocal space. Its normalized form ((3.15) and (3.25)) emphasizes how the energy spectrum (dependence of ${\tilde{w}}_{n}$ on *q* or r) transforms or gets reshaped in the material at various spatial positions *n*.

We may now introduce a single describer of quasi-static energy-dispersive properties of periodic materials, a *spectral entropy of deformation*, inspired by Shannon's definition (1.4) and using the normalized energy distribution ${\tilde{w}}_{n}(q,r)$ from (3.15),

4.1 $$H_{n}=-\sum_{q,r}{\tilde{w}}_{n}(q,r)\;\ln\;{\tilde{w}}_{n}(q,r)$$

and

4.2 $$q=\frac{2\pi \mu }{M},\;r=\frac{2\pi \ell }{L},\;\mu =0,1,\ldots M-1,\;\ell =0,1,\ldots ,L-1.$$

Also, a *normalized* spectral entropy can be introduced

4.3 $$h_{n}=\frac{1}{\ln\;ML}H_{n}=-\sum_{q,r}{\tilde{w}}_{n}(q,r)\;\ln\;{\tilde{w}}_{n}(q,r).$$

As a discrete function of *n*, the normalized entropy $h_{n}$ varies only from 0 to 1, which is very practical. Indeed, the zero corresponds to a Kronecker delta distribution with a single Fourier harmonic in the spectrum, and the unit value corresponds to a uniform distribution where all the harmonics contribute equally.

For the *plate-like lattice models*, entropy calculations use the normalized energy (3.25), based on (3.22)

4.4 $$H_{n}=-\sum_{q}{\tilde{w}}_{n}(q)\;\ln\;{\tilde{w}}_{n}(q)$$

and

4.5 $$h_{n}=-\frac{1}{\ln\;M}\sum_{q}{\tilde{w}}_{n}(q)\;\ln\;{\tilde{w}}_{n}(q).$$

The spectral entropy ((4.4) and (4.5)) is identical to a numerical spectral entropy introduced in [32] for a discretized two-dimensional elastic continuum. The same reference also provides exact analytical forms of the spectral entropy for the continuum material, loaded with Gauss-type distributed and concentrated traction forces. For the case of lattice materials, analytical results are not expected, but the forms (4.3) and (4.5) are generally applicable for *numerical* calculations of the strain energy spectral entropy in arbitrary periodic materials.

## 5. Raleigh wave solution over periodic half-space domains

Predictive design of strain energy transformation in specific lattice materials can be facilitated, using the fact the governing equation (2.3) or (3.16) allows for a static Raleigh wave fundamental solution

5.1 $$\lambda^{n}(q,r)\text{h}(q,r){\text{e}}^{\text{i}(qm+rl)}$$

and

5.2 $$\lambda^{n}(q)\text{h}(q){\text{e}}^{\text{i}qm}.$$

This solution represents a sinusoidal pressure wave of with an amplitude $\lambda^{n}$ decreasing in the material interior (λ<1), and, therefore, it is called an *exponential decay mode* solution [22,31,33]. Consider a figure 1 type problem with periodic boundary conditions, where the material sample is subjected to a distributed load at the left end (*n *= 0) and to a smooth reaction at the right end. Then, substituting (5.1) and (5.2) into (2.3) and (3.19) and using f=0 at *n* > 0, we find that the Raleigh wave ((5.1) and (5.2)) can be a valid solution, but only if the decay parameter λ satisfies the characteristic equations

5.3 $$\det\text{Z}(q,r)=0,\text{Z}(q,r)=\sum_{n}\lambda^{n}(q,r){\tilde{\text{K}}}_{n}(q,r)$$

and

5.4 $$\det\text{Z}(q)=0,\text{Z}(q)=\sum_{n}\lambda^{n}(q){\tilde{\text{K}}}_{n}(q),$$

and the vector h belongs to the nullspace of the characteristic matrix **Z**,

5.5 Z(q,r)h(q,r)=0

and

5.6 Z(q)h(q)=0.

A linear-polynomial solution, a⋅n+b, where a and b are constant vectors of the same size as d, will also satisfy the governing equations ((2.3) and (3.19)) with f=0. However, such a solution is not interesting for studying strain energy transformation in lattice materials, because it may only represent a uniform extension, a uniform shear and a rigid-body displacement of the entire lattice.

Since the force matrices ${\tilde{\text{K}}}_{n}$ (3.9) and (3.27), used in (5.3) and (5.4), represent *direct* interactions between the nodes in the lattice structure, they are non-zero for a small range of *n*, such as n=−1,0,1, when these interactions are limited to the nearest groups of nodes in the n-direction. Therefore, evaluation of an entire spectrum of the decay parameters λ(q,r) or λ(q) (for all *q* and r) is straightforward using (5.3) or (5.4). A linear superposition of the modes ((4.2), (4.3)) can be constructed to satisfy an arbitrary displacement or force boundary condition at n=0 [22,31]. Therefore, the *decay spectrum* λ(q,r) *or* λ(q) *determines how the strain energy ((3.11) and (3.21)) and entropy ((4.3) and (4.5)) of deformation translates into the material volume at n* > 0.

## 6. Problem statement and a continuum reference

**Figure 3.**
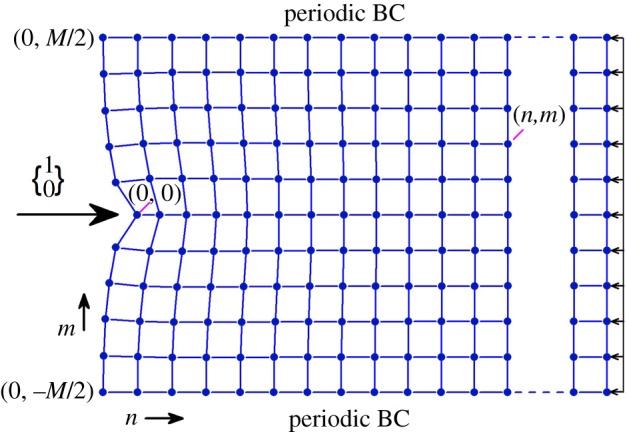
Problem statement with plane lattices of figure 4 type. The lattice is loaded with a unit point load at the left edge and balanced by a smoothly distributed load on the right edge. The loaded node is selected as the origin where the lattice indices, n=m=0. Periodic boundary conditions are applied along the horizontal edges making the lattice is *M*-periodic in the direction of the index *m*. (Online version in colour.)

**Figure 4.**
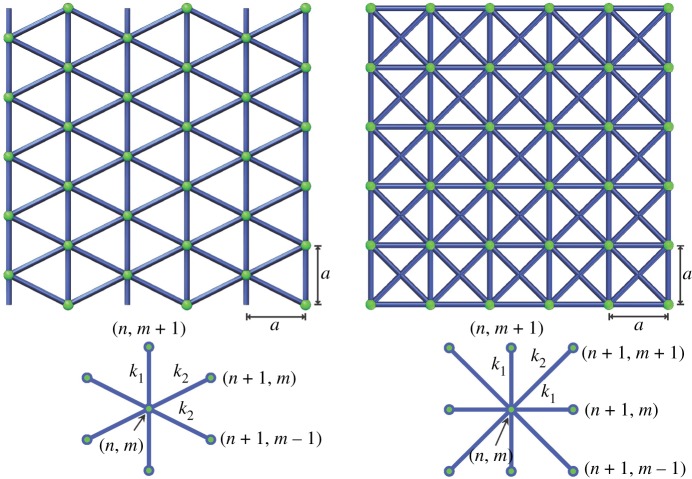
The triangular and X-braced plane lattices used in figure 3 problem statement, and their associate cells. For a proper comparison of strain energy distributions, the lattice constant (*a*) is made identical for both lattices and in both directions, i.e. the triangles in the first lattice are not equilateral. Only one design parameter, a ratio of the member stiffnesses $\kappa =k_{2}\text{/}k_{1},$ determine the shape of the Raleigh spectrum. (Online version in colour.)

A main objective of this work is to demonstrate opportunities for unusual strain energy transformation in highly non-local metamaterials. For this purpose, we consider a boundary value problem of figure 3 type with two different lattice materials, the triangular and X-braced ones, as described in figure 4. The lattice is loaded with a unit point load at the left edge and balanced by a smoothly distributed load on the right edge (figure 3). We select the point load, because it creates the narrowest possible strain energy concentration at the boundary, or its widest possible *spectral* distribution, where all Fourier harmonics are nearly equally represented. Since every harmonic *q* has its own decay rate λ(q), determined by the Raleigh spectrum (5.4), the initial strain energy distribution at the boundary will *transform* in the direction of the lattice index *n* pointed away from the boundary and towards the material interior. We will investigate this transformation by calculating the normalized strain energies $w_{nm}$ and ${\tilde{w}}_{n}(q)$ from (3.24) and (3.25), based on lattice deformation, and analysing their contour plots in the coordinates (n,m) and (n,q), respectively. Consequently, variance of Shannon's entropy of deformation (4.5) with distance to the load will also be prescribed by the Raleigh spectrum of the periodic material.

Referring to figure 3, a $w_{nm}$ data plot can be interpreted as transformation of the strain energy distribution vertically in space with a horizontal distance a⋅n to the point load, where *a* is a lattice constant. In turn, the ${\tilde{w}}_{n}(q)$ plots will show how the strain energy *spectral* distribution (over the wavenumber q) transforms with the distance a⋅n to the load.

*Continuum reference.* For a two-dimensional isotropic *elastic continuum* material in a state of plane stress or plane strain, Karpov [22] discussed a Raleigh wave solution, analogous to (4.2),

6.1 $$\lambda^{x}(q)\text{h}(q){\text{e}}^{\text{i}qm},$$

where the discrete lattice index *n* is replaced with a continuous material coordinate *x*, and showed that the corresponding decay spectrum is simply [22]

6.2 $$\lambda (q)={\text{e}}^{-|q|}.$$

Also, Karpov & Danso [32] developed analytical expressions for the strain energy densities and Shannon's entropy of deformation for homogeneous continuum materials. When the continuum domain is *L*-periodic in the direction for the vertical coordinate, like the lattice in figure 3, and is loaded with a concentrated unit force at the origin (x=y=0) normal to the boundary line (x=0), the following analytical results are valid [32]:

6.3 $$\begin{array}{rl} w_{L}(x,y) & =\frac{({\text{e}}^{4\pi x/L}-1)\text{/}L}{1+{\text{e}}^{4\pi x/L}-2{\text{e}}^{2\pi x/L}\cos2\pi y\text{/}L}, \end{array}$$

6.4 $$\begin{array}{rl} {\tilde{w}}_{L}(x,q) & =\frac{(2-\delta_{q0}){\text{e}}^{-2|q|x}}{2\coth2\pi x\text{/}L-1},\;q=\frac{2\pi \mu }{L},\;\mu =0,\pm 1,\ldots ,\pm M\text{/}2 \end{array}$$

6.5 $$\begin{array}{rl} \;\text{and}\;S_{L}(x) & =-\frac{4\;\ln\;2}{3+{\text{e}}^{4\pi x/L}}+\;\ln\;\left(\frac{2\coth2\pi x}{L}-1\right)+\frac{8\pi x\text{/}L}{1-\cosh4\pi x\text{/}L+2\sinh4\pi x\text{/}L}. \end{array}$$

**Figure 5.**
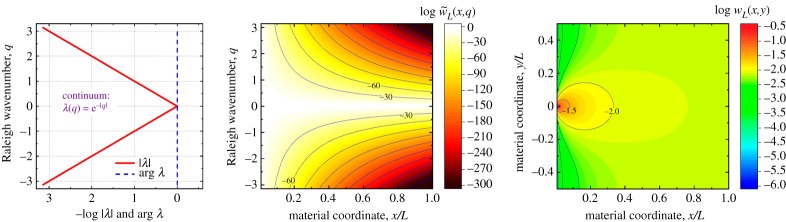
Raleigh spectrum (6.2), strain energy spectral (7.2) and volumetric (7.1) densities for a homogeneous elastic continuum in a state of plane stress or strain, due a concentrated unit load as in figure 3 problem. This basic reference data is compared with the analogous quantities in lattice materials in figures 6 and 7. (Online version in colour.)

Here, periodic boundary conditions are applied at y=±L/2. We may assume that L=a⋅M, where *a* is the lattice constant and *M* is a dimensionless integer number, the number of vertically repeating nodes in figure 3 lattice. Then, numerical calculations ((3.24), (3.25) and (4.5)) of the strain energy and entropy behaviour in lattice materials can be directly compared with (6.3)–(6.5), viewed as the continuum reference case. Appendix A also shows asymptotic cases of the strain energy distributions (6.3) and (6.4) and Shannon's entropy (6.5) for an infinitely large domain (L→∞), or for a load vicinity (x≪L), which may be useful in practice. We plot the continuum Raleigh spectrum (6.2) and energy distributions (6.3) and (6.4) in figure 5 for the comparisons to follow in §6.

## 7. Examples of strain energy transformation patterns

In this section, we demonstrate interesting unexpected behaviours and properties of strain energy distributions and spectral entropy in specific two-dimensional lattice materials, revealed by systematic analysis of their Raleigh spectra. Consider two types of two-dimensional periodic materials, the triangular and X-braced lattices of figure 4, loaded with a unit point load at their left edge and balanced by a smoothly distributed load on the right edge (figure 3). The triangular lattice of figure 4 is the simplest rigid plane lattice, which is also statically indeterminate [36]. Therefore, a pin-joined version is justified, since response of this lattice to all types of loads, except for couple moments concentrated at nodes, is dominated by axial deformation of the internodal links. The X-braced lattice is highly interesting in the area of mechanical metamaterial, being the first lattice, where reversal of Saint–Venant's edge effect was predicted [22]. Below, we will see that this behaviour is also accompanied by unusual pathways of the strain energy transformation in the lattice. The point load, as in figure 3, is interesting since it represents a quasi-static version of a broad-spectrum concentrated impact load. Strain energy spectrum due to such a load is flat initially, at the material surface, where all the Raleigh modes are present about equally [31]. In other terms, the point load induces the richest possible strain energy spectrum comprising the Raleigh wave modes ((5.1) and (5.2)).

Matrices of force constants for the pin-joined triangular lattice are

7.1*a* $${\text{K}}_{-11}={\text{K}}_{1-1}=-\frac{k_{2}}{5}\left[\begin{array}{cc} 4 & -2\\ -2 & 1 \end{array}\right],{\text{K}}_{-10}={\text{K}}_{10}=-\frac{k_{2}}{5}\left[\begin{array}{cc} 4 & 2\\ 2 & 1 \end{array}\right]$$

and

7.1*b* $${\text{K}}_{01}={\text{K}}_{0-1}=-k_{1}\left[\begin{array}{cc} 0 & 0\\ 0 & 1 \end{array}\right],{\text{K}}_{00}=2\left[\begin{array}{cc} 8k_{2}\text{/}5 & 0\\ 0 & k_{1}+2k_{2}\text{/}5 \end{array}\right],$$

and for the X-braced lattice

7.2*a* $$\begin{array}{rl} {\text{K}}_{-11} & ={\text{K}}_{1-1}=-k_{2}\left[\begin{array}{cc} 1 & -1\\ -1 & 1 \end{array}\right],{\text{K}}_{-1-1}={\text{K}}_{11}=-k_{2}\left[\begin{array}{cc} 1 & 1\\ 1 & 1 \end{array}\right], \end{array}$$

7.2*b* $$\begin{array}{rl} {\text{K}}_{01} & ={\text{K}}_{0-1}=-k_{1}\left[\begin{array}{cc} 0 & 0\\ 0 & 1 \end{array}\right],{\text{K}}_{10}={\text{K}}_{-10}=-k_{1}\left[\begin{array}{cc} 1 & 0\\ 0 & 0 \end{array}\right] \end{array}$$

7.2*c* $$\begin{array}{rl} \;\text{and}\;{\text{K}}_{00} & =2\left[\begin{array}{cc} k_{1}+2k_{2} & 0\\ 0 & k_{1}+2k_{2} \end{array}\right]. \end{array}$$

and all other ${\text{K}}_{nm}$ for these lattices are trivial. Here, $k_{1}$ and $k_{2}$ are elastic stiffnesses of the vertical/horizontal and inclined members, respectively.

**Figure 6.**
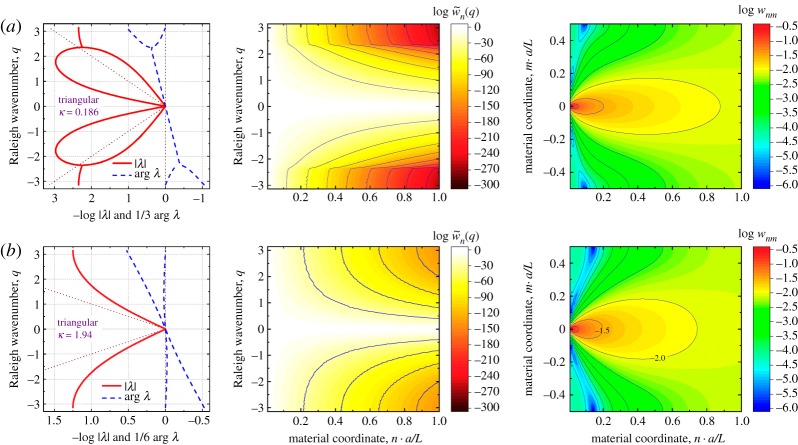
Two basic types of Raleigh decay spectra (5.4), strain energy spectral (3.25) and volumetric (3.24) densities for the triangular lattice, figure 4, subjected to a unit load at node (0,0), as shown in figure 3. Transition between the heart-shaped (*a*) and V-shaped (*b*) spectra occurs at κ=1.25. (Online version in colour.)

**Figure 7.**
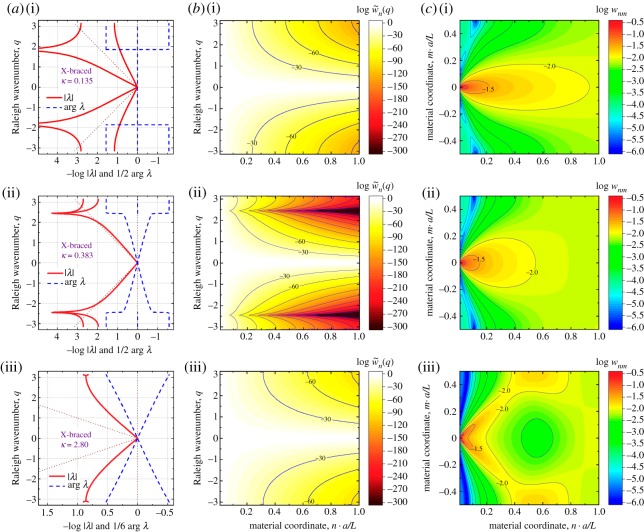
Three basic types of Raleigh decay spectra (5.4), strain energy spectral (3.25) and volumetric (3.24) densities for the X-braced lattice, figure 4, subjected to a unit load at node (0,0), as shown in figure 3. Two branches of real-valued decay parameters in the Raleigh spectrum (as on *a*(i)) occurs for values κ<0.25. Then with an increase of κ, these branches start to merge for smaller *q* and gradually form a simultaneous asymptotic bandgap at κ=0.383, as seen on the *b*(i). Further increase of the design parameter κ shifts the asymptotes away and turns the spectrum into the edge-split V-shape shown in *c*(i). Any further increase of κ only decreases the splitting, and the overall V-shape of the spectrum persists to dominate. The dark peak-shaped areas in *a*(ii),*b*(ii),*c*(ii) reflects filtering out Raleigh ways harmonics around q=±2.5 from the strain energy spectrum in the X-braced lattice, which contradicts the classical Saint–Venant principle. *c*(iii) Redirection of the strain energy maxima away from the horizontal load axis (figure 3) for an angle 40–45°. (Online version in colour.)

The matrices (7.1) and (7.2) are used to calculate numerically the Fourier transforms ${\tilde{\text{K}}}_{n}(q)$ and the decay spectra λ(q) plotted in figures 6 and 7, using equations (3.27) and (5.4) for all specific discrete values of the wavenumber parameter, q=2πμ/M, μ=0,1,…,M−1. The nodal displacement vectors, ${\text{d}}_{nm}$, are evaluated using a direct stiffness matrix method. In all cases discussed below, the lattice size is 4M×M with M=128 periodic nodes, the node (0,0) is constrained (${\text{d}}_{00}=0$) to provide a reference point for the displacements, and periodic boundary conditions are applied along the horizontal edges of the material model. Discrete Fourier transform, ${\tilde{\text{d}}}_{n}(q)$ of these nodal solutions are calculated from ${\text{d}}_{nm}$ according to (3.26). The displacement solution and its Fourier transform are used to calculate the normalized strain energy volumetric distribution (3.24), strain energy spectral distribution (3.25) and entropy of deformation (4.5). Below we discuss several interesting cases of the decay spectra, λ(q), energy distributions and entropy behaviour for various relative stiffnesses

7.3 $$\kappa =\frac{k_{2}}{k_{1}},$$

where $k_{1}$ and $k_{2}$ are the constants defined in equations (7.1) and (7.2).

The *triangular lattice* can demonstrate only two qualitatively different shapes of the Raleigh decay spectrum, depending on the value κ by equation (7.3), the ‘heart’ shape and the ‘V’ shape, whose examples are shown in figure 6. The Raleigh spectrum, as a dependence of the decay parameter λ on the wavenumber *q* was calculated using the condition (5.4). The heart- and V-shaped spectra obtained here represent two distinct patterns of strain energy transformation in the material. The difference in the pattern is well seen in the spectral distributions ${\tilde{w}}_{n}(q)$ of strain energy calculated by equation (3.25): the heart-shaped spectrum filters out higher harmonics more efficiently, and the contour lines flatten at higher values of the Raleigh wavenumber. However, the V-shaped spectrum gives a spectral distribution similar qualitatively to the continuum (figure 3), where the decay rate is a smooth and monotonous function of the wavenumber; the only difference is that the dependence λ(q) is slower than that in the continuum, and therefore higher harmonics generally propagate deeper into the material volume. The volumetric distributions $w_{nm}$ of the strain energy calculated by equation (3.24) do not show qualitative differences for the heart-shaped versus V-shaped spectra. However, the both spectra produce small ‘side pockets’ of low strain energy seen in deep blue tones at distance 0.1–0.15 n⋅a/L form the material surface, which is qualitatively different to the continuum case. Also, both volumetric distributions generally smoothen slower with distance to the surface than in the continuum.

Availability of the ‘side pockets’ in the volumetric distributions is the most surprising feature of the triangular lattice behaviour (figure 6). Another interesting observation is that the spectral density contours follows the lower branch of the heart-shaped spectrum, being a logical consequence of a smaller influence of the top branch comprised fast decaying modes. When the bottom branch merges with the top one (after q=2.4 and before q=−2.4), the decay rate λ(q) becomes nearly independent of *q*, leading to a selective filtering of the Raleigh waves. A triangular lattice with κ=0.186 is a low-pass filter of the Raleigh waves with a well-defined cut off wavenumber of about ±2.3.

The *X-braced lattice* can demonstrate even more interesting strain energy transformation properties. There are *three* distinct shapes of the decay spectrum, depending on the value κ by equation (7.3), figure 7, with a single-branch bandgap, dual-branch bandgap, and the V-shaped spectrum. In the first case, an asymptotic bandgap [22] occurs only on the top branch. Since energy spectral distribution follow the bottom edge of the spectrum, this bandgap does influence notably the energy distribution due to the concentrated load, and the spectral distribution resembles the continuum one of figure 5, in general.

We note that all cases considered so far, the continuum, triangular lattice heart-shaped and V-shaped spectra, give energy distributions consistent with Saint–Venant's principle, where at least the bottom branch of the Raleigh spectrum is a monotonously increasing function of the wavenumber. As a result, any higher wavenumber harmonics decays faster with the material coordinate than the lower (coarser) harmonics. This trend is violated for the dual-branch bandgap spectrum of the X-braced lattice (figure 7*a*(ii),*b*(ii),*c*(ii)). The spectral energy contours, as usually, follow the bottom branch of the spectrum, and since the bandgap occurs simultaneously at both branches, the harmonics around q=±2.4 are entirely absent from the spectral distribution even at small distances to the load. This absence is seen as the peak-shaped (dark tone) regions on the contour plot ${\tilde{w}}_{n}(q)$. These regions (centered around q=±2.4) represent very fast decaying modes, while modes with |q|>2.4 may decay much slower, which contradicts Saint–Venant's principle. This is an occurrence of Saint–Venant's principle reversal first predicted in [22], and the contour plot ${\tilde{w}}_{n}(q)$ of figure 7*a*(ii),*b*(ii),*c*(ii) offers a clear graphical illustration of this interesting phenomenon. Thus, the X-braced lattice with κ=0.383 is a mechanical metamaterial reversing Saint–Venant's edge effect principle, and in practice it may also be viewed as a selective filter of Raleigh pressure waves with wavenumbers close to ±2.4.

The last example shown in figure 7*a*(iii),*b*(iii),*c*(iii) is a V-shaped decay spectrum occurring for the X-braced lattice at κ=2.80. This spectrum type and the corresponding contour plot ${\tilde{w}}_{n}(q)$ may look similar to the V-shaped spectrum case of the triangular lattice, figure 6. However, the *volumetric* energy distribution offers a surprise. As can be seen, the lattice contains a large region of lower strain energy in the direction of the surface load, seen there in green tones. Most importantly, areas of maximal strain energy have elongated shapes oriented at angle 40–45° to the direction of (horizontal) surface load. Thus, we observe a phenomenon *strain energy deflection* or *redirection* from the load axis. The V-shaped Raleigh spectrum, which a plot of |λ(q)|, gives no hint about this behaviour, unless we consider also the *complex phases* of the parameters λ(q). For the V-shaped spectrum of the continuum, figure 5, we see that arg⁡λ=0 for all *q*, meaning that the entire decay spectrum happened to be real-valued. For the V-shaped spectrum of the triangular lattice, figure 6*b*, the decay parameters ($\lambda_{1}$ and $\lambda_{2}$) from the two branches have equal absolute values but entirely different complex phases. By contrast, for the V-shaped spectrum of the X-braced lattice, figure 7*a*(iii),*b*(iii),*c*(iii), $\arg\lambda_{1}=-\arg\lambda_{2}$, i.e. all the decay parameters come in complex conjugate pairs at every given wavenumber *q*. As result, we observe a special form synergy or collective behaviour of the Raleigh modes in the X-braced lattice: two Raleigh waves (6.1) with complex conjugate lambdas shift the locations of maximal deformation in the opposite vertical direction, while the middle region of the lattice is experiencing a mutual cancelling of these waves.

This last observation of strain energy redirection away from the axis of surface load, figure 7*a*(iii),*b*(iii),*c*(iii), due to a synergetic phase shift and self-cancelling of complex conjugate Raleigh waves, is the most interesting result of the present study.

**Figure 8.**
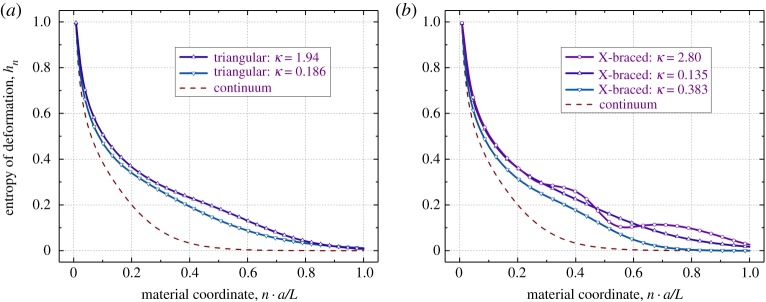
Behaviour of Shannon's spectral entropy of deformation (4.5) in the triangular (*a*) and X-braced (*b*) lattices, compared to the continuum case (6.5), for the concentrated load problem of figure 3 type. Different values of the design parameter κ from equation (7.3) are used, as in figures 6 and 7 data. (Online version in colour.)

We finally note behaviour of Shannon's spectral entropy of deformation (4.5) for the triangular and X-braced lattices, calculated from the energy spectral distribution data of figures 6 and 7, and compared with the continuum case (6.5) (figure 8). As can be seen, the continuum entropy is monotonous and convex for all *x*. However, in the lattices it may exhibit more complex behaviours depending on the design parameter κ defined in (7.3). We found that the profile of entropy in the triangular lattice exhibits flexure points, seen in figure 8, when κ>0.63; although no local maxima or minima are possible at all. The X-braced lattice shows flexure points when κ>0.165, as well as local maxima and minima once κ>0.469. Interestingly, the phenomenon of strain energy redirection, mentioned above in this section, is seen *together* with the occurrence of these local maxima and minima in the X-braced lattice, i.e. when the value of κ is greater than 0.469. The triangular lattice shows no stationary points on its entropy profile, and logically, no energy redirection behaviour.

Thus, there is a general tendency for the spectral entropy to decay slower in lattices than in the continuum, a manifest of the more complex, spectrally richer deformation of the lattice materials. In highly non-local and highly anisotropic lattices, the entropy of deformation, as function of distance to loads, may have flexure points and even local maxima, which may signify highly unusual mechanical behaviours. However, in a ‘no surprise’ elastic continuum, the spectral entropy is monotonous and convex.

## 8. Conclusion

We have discussed several non-standard analytical tools that could facilitate a systematic analysis of arbitrary periodic materials: associate substructure, Raleigh wave solution and decay spectrum, Parseval's energy theorem, strain energy spectral density and Shannon's entropy of deformation. We showed that the *Raleigh spectrum is the main material's characteristic which fully determines its strain energy transformation behaviour*. Considered examples of the triangular and X-braced lattices showed that there are only a few distinct types of the Raleigh spectra for a given material, depending on its design parameters, making qualitative prediction of its strain energy transformation behaviours systematic and comprehensive. Surprising *anomalous behaviours* were observed for these relatively simple lattices: (i) selective Raleigh wave filtering (surface blockage), (ii) reversal of Saint–Venant's edge effect, (iii) formation of ‘hiding’ side pockets of low deformation, and most interestingly (iv) strain energy redirection away from the axis a of concentrated surface load. This last phenomenon arises from a synergetic phase shift and self-cancelling of complex conjugate Raleigh wave pairs in a highly non-local mechanical metamaterial. The energy redirection effect is signified by appearance of stationary points in the spatial profile of the strain energy spectral entropy of Shannon's type, introduced here as a measure of complexity of mechanical deformation. Strain energy redirection provides practical opportunities for impact energy harnessing and managing earlier stages of shock wave propagation in mechanical and acoustical metamaterials, even before any oscillatory motion or wave packet propagation is established.

## Data accessibility

This article does not contain any additional data.

## Appendix A

Asymptotic cases of the strain energy distributions and Shannon's entropy of deformation for an infinitely large domain (L→∞), or for a close proximity to the load (x≪L), adapted from [32]:

A 1 $$\begin{array}{r} w_{0}(x,y)=\frac{x}{\pi (x^{2}+y^{2})}, \end{array}$$

A 2 $$\begin{array}{r} {\tilde{w}}_{0}(x,q)=x{\text{e}}^{-2|q|x} \end{array}$$

A 3 $$\begin{array}{r} \;\text{and}\;S_{0}(x)=1-\;\ln\;x. \end{array}$$

Relationships between (A 1)–(A 3) and (7.1)–(7.3):

A 4 $$\begin{array}{r} w_{0}(x,y)=\lim_{L\to \infty }w_{L}(x,y), \end{array}$$

A 5 $$\begin{array}{rl} {\tilde{w}}_{0}(x,q) & =\frac{1}{2\pi }\lim_{L\to \infty }L\cdot {\tilde{w}}_{L}\left(\frac{x,qL}{2\pi }\right) \end{array}$$

A 6 $$\begin{array}{rl} \;\text{and}\;S_{0}(x) & =\lim_{L\to \infty }\left(S_{L}(x)-\;\ln\;\frac{L}{2\pi }\right). \end{array}$$
